# Sexual dysfunction in women with multiple sclerosis: prevalence and impact on quality of life

**DOI:** 10.1186/s12894-020-0581-2

**Published:** 2020-02-21

**Authors:** Fatemeh Nazari, Vahid Shaygannejad, Mehrdad Mohammadi Sichani, Marjan Mansourian, Valiollah Hajhashemi

**Affiliations:** 1 Isfahan neurosciences Research center, Isfahan University of Medical Sciences, Isfahan, Iran; 2 Department of Adult Health Nursing, Faculty of Nursing and Midwifery, Isfahan University of Medical Sciences, Isfahan, Iran; 3 Department of Neurology, School of Medicine, Isfahan University of Medical Sciences, Isfahan, Iran; 4 Isfahan Kidney Transplantation Research Center, Isfahan University of Medical Sciences, Isfahan, Iran; 5 Department of Urology, School of Medicine, Isfahan University of Medical Sciences, Isfahan, Iran; 6 Department of Epidemiology & Biostatistics, School of Health, Isfahan University of Medical Sciences, Isfahan, Iran; 7 Isfahan Pharmaceutical Sciences Research Center, Isfahan University of Medical Sciences, Isfahan, Iran; 8 School of Pharmacy and Pharmaceutical Sciences, Isfahan University of Medical Sciences, Isfahan, Iran

**Keywords:** Sexual dysfunction, Prevalence, Multiple sclerosis, Women

## Abstract

**Background:**

Sexual function is a component of quality of life, and sexual dysfunction entails reduced satisfaction with life and impaired mood and quality of relationships and affects not only the individual’s quality of life, but her partner’s life as well. Since Sexual Dysfunction (SD) is among the most common complaints of patients with Multiple Sclerosis (MS), this study was conducted to determine the prevalence of SD among women with MS and its effect on quality of life.

**Materials and methods:**

This cross-sectional study was conducted in 2018 on 300 women with MS aged 22–50 years in Isfahan, Iran, selected through systematic random sampling. Data were collected using the standard Female Sexual Function Index (FSFI) and MSQOL-54 and analyzed in SPSS using descriptive and analytical statistics.

**Results:**

The overall prevalence of SD was found as 69.8% in women with MS, with the dimension of sexual desire being affected in 38.6% of the cases, sexual arousal in 38.6%, lubrication in 23.7%, orgasm in 37.3%, satisfaction in 23.4% and pain in 16.9%. SD was found to have significant relationships with age, duration of marriage, fatigue, EDSS and the combined physical and mental health aspects of quality of life (*P* <  0.05). Moreover, logistic regression analysis revealed that there was a higher probability of a sexual dysfunction among patients with MS and a high fatigue score [1.228(1.003 to 1.504); *P* = 0.047]. The mean score of the combined physical and mental health aspects of quality of life was lower in the group of women with MS and SD compared to those without SD, and the difference between the two groups was statistically significant (*P* <  0.05).

**Conclusion:**

Sexual dysfunction is highly prevalent among women with MS and affects various dimensions of quality of life. Greater attention should be paid to the sexual problems faced by MS patients in order to improve their quality of life.

## Background

Multiple Sclerosis (MS) is a chronic and degenerative inflammatory disease that is identified by the demyelination of the nervous system [1] and affects women three times more than men [2]. Sexual dysfunction is a common feature of MS and one of the main factors contributing to distress [3]. Most MS patients (even those with mild degrees of disability) experience various sexual disorders that affect different aspects of their life significantly, which demonstrates the importance of the early identification and treatment of these disorders [4]. Sexual dysfunction due to MS is more prevalent in women than men, varying from 40 to 80% [5]. Many studies, including ones by Ghajarzadeh (2013), Merghati Khoei (2013) and Mohammadi (2008), have reported the prevalence of SD in Iranian women with MS as 66, 87.1 and 55.3%, respectively [6–8]. The main sexual problems of women with MS include the loss of libido, impaired orgasm, reduced vaginal lubrication and the loss of genital sensation [6, 9, 10].

The nature of SD in MS patients is complex and its cause is debatable [3, 11]. There seems to be a complex interaction among social, physiological and psychological factors that is strongly affected by emotions and social elements [12]. MS patients have poorer quality of life compared to healthy people, and sexual function is one of the key factors affecting their quality of life that can lead to great tension [10, 13, 14]. MS commonly occurs between ages 20 and 40 years, when the patients are normally sexually active and at the peak of their personal and family responsibilities and at a time when they often plan to build a family and establish intimate relationships; consequently, sexual problems may cause a greater decline in the quality of life of this group [13, 15, 16]. Sexual function is a component of quality of life, and sexual dysfunction entails reduced satisfaction with life and impaired mood and quality of relationships [16] and affects not only the individual’s quality of life, but also her partner’s [17]. Previous studies have shown that the identification and treatment of these problems improve couples’ quality of life [17, 18].

Despite the prevalence of SD in MS patients, 63% of the patients reported that they had never talked to physicians or healthcare providers about their sexual problems [19]. In Iran, it is not easy to discuss sexual problems due to cultural and religious reasons [20], and cultural barriers have been identified as a major challenge to proper sexual functioning [21]. Given the importance of sexual health for improving the quality of life and satisfaction with it [20] and considering that sexuality affects certain aspects of life significantly, such as mental image, self-esteem and interpersonal and marital relationships [3], sexual function needs to be further studied [22]. In view of the very few studies conducted on the subject in Iran, and since medical personnel’s awareness about the prevalence of sexual dysfunction and its related factors can form the basis of interventions and new medical and counseling strategies for improving the quality of life in MS patients and preserving their family unity, this study was conducted to determine the prevalence of sexual dysfunction and its impact on quality of life in women with MS.

## Materials and methods

This cross-sectional study was conducted from Aug. 23, 2017, to April 19, 2018, on married women with MS visiting the neurology clinics of Kashani and Alzahra hospitals affiliated to Isfahan University of Medical Sciences, selected through systematic random sampling. Based on the results of a previous study on the prevalence of SD in MS patients [7], with *P* = 87.1%, confidence interval of 95% (1.96) and d = 0.038, the sample size was determined as 300.

The study inclusion criteria consisted of a definitive diagnosis of MS by a neurologist based on the revised McDonald criteria (2010) [23], age over 18 years, being a patient of the referral clinics, physical and mental ability to respond to the questions, being a resident of Isfahan, willingness to take part, being married, no acute attacks over the last month, no acute heart disease or diabetes and hypo- or hyperthyroidism and no other chronic diseases or any acute mental disease based on the patient’s self-report. The exclusion criterion was gynecological pathologies, pregnancy, not sexuality active during the last 6 months and unwillingness to take part in the study.

After the subjects completed informed consent forms, data were collected through interviews using a seven-part questionnaire. The first part contained four items on personal details (age, duration of marriage, education and occupation). The second part included five items on clinical conditions (disease duration, age at diagnosis, EDSS, clinical pattern of MS and medications used). The third part entailed the FSFI items; that is, 19 items assessing different dimensions of women’s sexual function, namely desire, arousal, lubrication, orgasm, satisfaction and pain over the last 4 weeks [23, 24]. In this index, the score of each item ranges from 1 to 5 points in the dimension of desire, and from 0 to 5 points in all the other dimensions. The total index score is the sum of the scores of all its six dimensions. The score of each dimension is found by the sum of the scores of all the items in that dimension, multiplied by its specific coefficient, as follows: Desire = 0.6, arousal = 0.3, lubrication = 0.3, orgasm = 0.3, satisfaction = 0.4 and pain = 0.4. The scores ranged from 1.2 to 6 points for the dimension of desire and from 0 to 6 points for all the other dimensions. The total scores of sexual dysfunction ranged from 1.2 to 36 points, and higher scores indicated better sexual function in the subject. The cut-off point was 3.3 for desire, 3.4 for arousal, 3.7 for lubrication, 3.4 for orgasm, 3.8 for satisfaction, 3.8 for pain and 28 for the overall FSFI [25]. The reliability of the FSFI was confirmed by Mohammadi (2008) in Iran and Rosen (2000) abroad [25, 26]. In the present study, the reliability of the FSFI was determined using the test-retest method on ten women with MS, over two stages with a ten-day interval, and the correlation between the two tests for FSFI was found as *r* = 0.81. The fourth part included the MSQOL-54 items, 18 of which measure 14 MS-specific domains and 36 of which deal with the general quality of life. All the questionnaire items are scored based on a 2- to 7-point Likert scale and measure the quality of life in the following 14 domains: Physical function, role limitation due to physical problems, role limitation due to emotional problems, social function, health distress, sexual function, satisfaction with sexual function, pain, energy, health perceptions, general quality of life, change in health, cognitive function and emotional well-being, plus physical and mental composites of health [27]. The scores in all the 14 domains, including the composite domains, range from 0 to 100 points, and higher scores suggest better quality of life. The physical health composite consists of eight domains and the mental health composite of five domains, and are assessed by calculating the weight percentage of each domain. Higher scores in all the domains indicate better conditions. The validity of the MSQOL-54 has been confirmed in various studies [28]. The fifth part of the study tool entails the nine items of the Fatigue Severity Scale (FSS), including five that measure the quality of fatigue more than its quantity (items 1, 2, 3, 4, and 6), three that measure physical and mental fatigue and the effect of fatigue on social status (items 5, 7, and 9) and one last item (8) that compares the severity of fatigue against the other symptoms in the MS patient. Each item is scored from 1 to 7, and 1 means ‘total disagreement’ and 7 ‘total agreement’. The total score is obtained by dividing the sum of the scores by nine, resulting in a score between 1 and 7, where 7 shows the highest fatigue level and 1 indicates the absence of fatigue [29]. The sixth part is the Depression, Anxiety, and Stress Scale (DASS-21) in which each cluster of 7 items measures one factor or emotional state in order to assess depression, anxiety, and stress. Each question is scored on a Likert scale ranging from 0 to 3. Ghafari et al. calculated the internal consistency of the DASS-21 using Cronbach’s alpha and the Cronbach’s alpha of the Subscales of depression, anxiety, and stress was 0.97, 0.71, and 0.74, respectively [30]. Another questionnaire used for assessing the severity of physical and neurological disability in MS patients was John Kurtzke’s Expanded Disability Status Scale (EDSS), which assesses the functional status of eight systems, including the pyramidal, cerebellar, brainstem, sensory, bowel and bladder, visual and cerebral regions, with scores from 0 to 10, where 0 shows normal neurological status and 10 indicates MS-induced death [31]. This scale was completed by the researcher under the supervision of a neurologist. Data were analyzed in SPSS-18 (SPSS Inc., Chicago, IL., USA) using descriptive (mean and SD and n %) and inferential (ANOVA, Pearson’s correlation coefficient and the independent t-test) statistics. The level of statistical significance was set at *P* <  0.05.

## Results

A total of 342 married women with MS over the age of 18 years took part in this study, including 42 (12.5%) without any sexual activity over the last 6 months and 300 (67.5%) with sexual activity. Table 1 presents participants’ demographic and clinical details (age, duration of marriage, education, occupation, disease duration, age at diagnosis, clinical pattern, EDSS, fatigue and medications used). Given the determined cut-off points, 211 (70.3%) of the women with MS scored less than 28 in the FSFI, and assessing the different dimensions of this index based on the cut-off points showed that 116 (38.7%) had dysfunctional desire, 116 (38.7%) dysfunctional arousal and 104 (34.7%) dysfunctional lubrication, 112 (37.3%) were unable to reach orgasm, 69 (23%) were sexually dissatisfied and 52 (17.3%) had pain during intercourse (Table 2).

**Table 1:** Demographic and clinical variables in the study subjects

Variable		Mean (±SD)	Minimum	Maximum
Age (years)		36.35 ± 7.33	22.00	50.00
Duration of marriage (years)		15.07 ± 8.55	0.16	33.00
Disease duration (years)		7.37 ± 5.40	0.02	26
Age at diagnosis (years)		27.92 ± 7.57	11.00	49.50
EDSS		2.06 ± 1.85	0.00	7.50
Fatigue		3.65 ± 1.57	1.00	7.00
Anxiety		13.18(9.18)	0	20
Depression		13.46(9.57)	0	28
Stress		19.59(10.03)	0	34
QOL	Physical health composite score	62.66 ± 19.15	15.42	96.24
	Mental health composite score	60.75 ± 19.99	9.07	97.34
Education N (%)	Secondary school or below	74 (24.7)		
	High school	132 (44.0)		
	University	94 (31.3)		
Occupation N (%)	Housewife	258 (86.0)		
	Employed	33 (11.0)		
	Retired	9 (3.0)		
Disease pattern N (%)	Relapsing-Remitting MS (RRMS)	243 (81.0)		
	Progressive MS	39 (13.0)		
	Clinically Isolated Syndrome (CIS)	18 (6.0)		
Taking medications N (%)	No	30 (10.0)		
	Yes 270 (90.0)	Disease Modifying Therapy (DMT)		141 (47.0)
		Anticholinergic drugs		51 (17.0)
		Psychiatric drugs		8 (2.7)
		Antidepressants drugs		56(18.7)
		Benzodiazepines drugs		18(6.0)
		Anticonvulsants drugs		50(16.7)
		Beta blockers drugs		17 (5.7)
		Antispasmodic drugs (baclofen,tizanidine, Methocarbamol)		38(12.7)
		Complementary drugs		183 (61.0)

**Table 2:** The prevalence of sexual dysfunction and its mean score in married women with MS

Domain	Cut-Off Point	N (%) < Cut-Off Point	Mean (±SD)	Max/Min
Desire	3.3	116 (38.7)	3.41 (1.05)	6.00/1.20
Arousal	3.4	116 (38.3)	3.61 (1.18)	6.00/1.20
Lubrication	3.7	104 (34.7)	4.28 (1.28)	6.00/1.20
Orgasm	3.4	112 (37.3)	3.76 (1.27)	6.00/1.20
Satisfaction	3.8	69 (23.0)	4.57 (1.16)	6.00/1.20
Pain	3.8	52 (17.3)	4.99 (1.18)	6.00/1.20
Total	28	211 (70.3)	24.64 (5.11)	34.40/7.20

Pearson’s correlation coefficient showed that the total FSFI score had an inverse and significant correlation with age (*P* = 0.004), duration of marriage (*P* = 0.004), fatigue (*P* ≤ 0.001) and EDSS (*P* = 0.004), but no significant relationship with age at onset of disease or the duration of the disease (*P* > 0.05). The scores of the different dimensions of sexual function were found to have inverse and significant correlations with age, fatigue and the duration of marriage (*P* <  0.05).

Age at onset of disease had inverse correlations with arousal (*P* = 80.01) and pain (*P* = 0.001), but no significant relationships were observed between this variable and the other dimensions (*P* > 0.05). Pearson’s correlation coefficient showed that the total FSFI score and its subscale scores (except for desire, lubrication, pain) had an inverse and significant correlation with depression (*P* <  0.05). No significant relationships between anxiety and the total FSFI score and its subscale scores (*P* > 0.05). Also, no significant correlation were found between stress and the total FSFI score and its subscale scores [except for desire(*r* = 0.114, *P* = 0.048)].

An inverse and significant correlation was found between disease duration and the lubrication scores (*P* = 0.011), but no significant relationships were observed between this variable and the other dimensions of sexual function (*P* > 0.05). The EDSS level also had inverse correlations with arousal (*P* = 0.010), lubrication (*P* = 0.028), orgasm (*P* = 0.003) and satisfaction (*P* = 0.008), but had no significant relationships with the desire and pain dimensions (*P* > 0.05). No significant differences were found between the mean total FSFI score and any of the subscales (except for lubrication) in terms of the clinical pattern of MS in the three groups, and the mean score of lubrication was less in the progressive group than the RRMS and CIS groups, but statistically significant differences were observed only between the mean scores of lubrication (*P* = 0.017) in terms of clinical pattern. Pearson’s correlation coefficient showed significant positive relationships between the total FSFI score and its subscale scores and physical and mental health composites (*P* ≤ 0.001).). Also, No significant differences were found between the mean total FSFI score and any of the subscales (except for Arousal) in terms of the education in the three groups, and the mean score of Arousal was less in the Secondary school or below group than the High school and University groups and statistically significant differences were observed only between the mean scores of lubrication (*P* = 0.010) in terms of education (Table 3).

**Table 3:** The relationship between the six domains of the FSFI and demographic and clinical variables in the study subjects

FSFI Domain		Desire	Arousal	Lubrication	Orgasm	Satisfaction	Pain	Total Sexual Dysfunction
Age	r	−0.212^**^	−0.238^**^	−0.119^*^	− 0.156^**^	− 0.154**	0.153**	− 0.166**
	p	< 0.001	< 0.001	0.040	0.007	0.008	0.008	0.004
Duration of marriage (years)	r	−0.171**	− 0.237**	− 0.150**	− 0.169**	−0.141*	0.153**	−0.166**
	p	0.003	< 0.001	0.009	0.003	0.015	0.008	0.004
Fatigue	r	−0.173**	−0.226**	−0.261**	− 0.234**	−0.252**	− 0.195**	−0.313**
	p	0.003	< 0.001	< 0.001	< 0.001	< 0.001	0.001	< 0.001
Age at diagnosis	r	−0.107	− 0.137*	0.021	− 0.049	−0.041	0.195**	−0.024
	p	0.065	0.018	0.711	0.397	0.475	0.001	0.673
Anxiety	r	0.070	−0.094	0.009	− 0.105	−0.093	0.008	−0.054
	p	0.229	0.104	0.879	0.069	0.109	0.887	0.349
Depression	r	0.007	−0.200**	−0.066	−0.147*	− 0.118*	−0.043	− 0.135*
	p	0.900	< 0.001	0.252	0.011	0.040	0.453	0.020
Stress	r	0.114^*^	−0.076	0.028	−0.075	− 0.060	−0.043	− 0.029
	p	0.048	0.189	0.627	0.195	0.301	0.454	0.613
Disease duration (years)	r	−0.069	−0.041	−0.147*	− 0.173**	−0.101	− 0.044	−0.106
	p	0.235	0.483	0.011	0.003	0.082	0.450	0.068
EDSS	r	−0.071	−0.148*	−0.127*	− 0.173**	−0.154**	− 0.024	−0.164**
	p	0.219	0.010	0.028	0.003	0.008	0.676	0.004
Physical health composite score	r	0.257^**^	0.368^**^	0.319^**^	0.323^**^	0.365^**^	0.195^**^	0.427^**^
	p	< 0.001	< 0.001	< 0.001	< 0.001	< 0.001	0.001	< 0.001
Mental health composite score	r	0.225**	0.300**	0.212**	0.290**	0.303**	0.193**	0.354**
	p	< 0.001	< 0.001	< 0.001	< 0.001	< 0.001	0.001	< 0.001
MS Pattern Mean (SD)								
CIS	18	3.46(0.76)	3.58(1.14)	5.01(0.86)	3.51(1.40)	4.97(1.18)	5.20(0.87)	25.75(4.18)
RRMS	243	3.42(1.06)	3.66(1.16)	4.27(1.26)	3.84(1.26)	4.57(1.13)	4.94(1.20)	24.73(5.17)
Progressives	39	3.35(1.09)	3.28(1.26)	4.02(1.47)	3.37(1.13)	4.35(1.29)	5.16(1.12)	23.56(5.02)
ANOVA	F	0.093	1.760	3.819	2.750	1.755	0.871	1.337
	P	0.911	0.174	0.023	0.066	0.175	0.420	0.264
Education Mean (SD)								
Secondary school or below	74	3.29(1.21)	3.25(1.28)	3.98(1.50)	3.46(1.36)	4.37(1.27)	5.23(1.18)	23.58(5.72)
High school	132	3.43(0.996)	3.72(1.21)	4.40(1.19)	3.89(1.23)	4.57(1.18)	4.95(1.15)	24.95(5.14)
University	94	3.50(.990)	3.74(0.99)	4.37(1.20)	3.82(1.21)	4.74(1.02)	4.86(1.22)	25.04(4.46)
ANOVA	F	0.811	4.685	2.844	2.866	2.029	2.097	2.113
	P	0.445	0.010	0.060	0.058	0.133	0.125	0.123

*P*-value resulted from Pearson’s test and ANOVA,

**. Correlation is significant at the 0.01 level (2-tailed),

*. Correlation is significant at the 0.05 level (2-tailed)

Moreover, variables that showed significant unadjusted associations with sexual dysfunction were fatigue, Body Mass Index (BMI). The final regression model showed that there was a higher probability of a sexual dysfunction among women with MS and a high fatigue score [1.228(1.003 to 1.504); *P* = 0.047] (Table 4).

**Table 4:** Multivariable Logistic regression analysis of women with multiple sclerosis and sexual dysfunction

Characteristic	Unadjusted odds ratio (95% CI)	P	Adjusted odds ratio (95% CI)	P*
Age (years)	1.032 (0.997 to 1.069)	0.073	1.133 (0.933 to 1.306)	0.085
Marriage duration (years)	1.020 (0.990 to 1.050)	0.193	0.981 (0.918 to 1.047)	0. 559
Fatigue	1.254 (1.064 to 1.478)	0.007	1.228 (1.003 to 1.504)	0.047
EDSS*	1.101 (0.957 to 1.267)	0.177	0.987 (0.788 to 1.235)	0.907
BMI**	1.071(1.004 to 1.143)	0.037	1.071 (0.998 to 1.149)	0.055
Depression	1.013 (0.986 to 1.040)	0.355	1.023 (0.973 to 1.076)	0.367
Anxiety	1.008 (0.981 to 1.036)	0.572	1.012 (0.962 to 1.065)	0. 638
Stress	0.999 (0.975 to 1.024)	0.938	0.971 (0.925 to 1.019)	0. 230
Disease duration(years)	1.015 (0.969 to 1.063)	0.540	0.916 (0.803 to 1.046)	0.195
Age at diagnosis(years)	1.011(9.979 to 1.045)	0.532	0.920(0.813 to 1.041)	0.184
Disease Modifying Therapy(DMT)	1.149(0.700–1.887)	0.583	1.002(0.574 to 1.749)	0.994
Antidepressants drugs	0.520(0.255–1.060	0.072	0.641(0.294 to 1.398)	0.264
Anticonvultion drugs	0.541(0.258 to 1.137)	0. 105	0.708(0.311 to 1.609)	0.410
Antispasmodic drugs	0.827(0.384 to 1.785)	0.629	1.099(0.440 to 2.745)	0.840
Course disease				
Relapsing remitting	0.878 (9.302 to 2.553)	0.812	0.806 (0. 328 to 1.979)	0. 638
Progressive	1.115 (.317 to 3.921)	0.865	0.807 (0.420 to 1.551)	0.520
CIS***	Ref			
education				
Secondary school or below	1.145(0.582–2.255)	0.694	0.806(0.328 to 1.979)	0.638
High school	0.942(0.530–1.674)	0.838	0.807(0.420 to 1.551)	0.520
University	Ref			

EDSS*, Expanded Disability Status Scale. BMI**, Body Mass Index. CIS***, Clinical Isolated Syndrome. OR adjusted based on individual variables (age, duration of marriage, BMI, education) and clinical variables (age of onset of disease, disease duration, EDSS, course disease, Fatigue, anxiety, depression, stress, Antidepressants drug, Disease Modifying Therapy (DMT), Antispasmodic and Anticonvultion drugs)

The mean scores of all the subscales of MSQOL were lower in the married women with MS and SD compared to those without SD, and significant differences were found between the two groups in all the dimensions, except for limitations due to emotional problems and change in health. Moreover, the scores of the physical and mental health composites of quality of life were significantly lower in the women with MS and SD compared to those without SD (*P* <  0.05) (Table 5).

**Table 5:** A comparison of the mean scores of quality of life (QOL) in women with MS with and without Sexual dysfunction (SD)

QOL Domain	Without SD (FSFI > 28)	with SD (FSFI < 28)	t	P
	Mean (SD)	Mean (SD)		
Physical function	71.85 (28.69)	60.68 (29.78(	2.998	0.003
Role limitation due to physical problems	54.49 (44.36)	43.36 (43.75)	2.004	0.046
Role limitation due to emotional problems	51.31 (47.95)	40.91 (45.37)	1.742	0.083
Pain	83.85 (21.30)	69.49 (24.72)	5.079	< 0.001
Emotional well-being	65.52 (18.61)	57.59 (21.13)	3.072	0.002
Energy	55.55 (20.32)	49.04 (21.15)	2.460	0.014
Health perceptions	60.84 (20.17)	55.59 (20.47)	2.038	0.042
Social function	82.49 (16.95)	72.59 (22.01)	4.212	< 0.001
Cognitive function	80.00(21.63)	70.40 (25.48)	3.323	0.001
Health distress	75.00 (23.60)	68.10 (27.47)	2.067	0.040
Sexual function	93.70 (13.70)	62.48 (26.09)	13.346	< 0.001
Change in health	51.40 (29.03)	47.81 (26.15)	1.046	0.297
Satisfaction with sexual function	85.17 (21.13)	55.76 (30.44)	9.466	< 0.001
Overall QOL	72.05 (15.69)	64.40 (18.40)	3.430	0.001
Physical health composite score	69.97 (17.02)	59.63 (19.20)	4.336	< 0.001
Mental health composite score	66.78 (19.13)	58.21 (19.85)	3.455	0.001

*FSFI* Female Sexual Function Index

## Discussion

Our study was designed to investigate prevalence and distribution of SD dimensions, and to identify contributory factors for SD in women with MS. The present findings support the results of previous studies, which have shown a high prevalence for sexual dysfunction among women with MS. The prevalence of SD was reported as 70.3% (*n* = 211) in the present study and as 27.27% by Bartnik et al. (2017), 55.6 by Marck et al. (2016), 82.5% by Lew-Starowics (2013), 63.5% by Ashtari et al. (2014) and 87.1% by Merghati-Khoei (2013) [1, 5, 7, 32, 33]. In a systematic Meta analytic review in Iran (2019), the prevalence of SD was reported as 62% in women with MS [34] and as 34 to 85% in a review study in Canada (2014) [35]. The difference in the results obtained may be due to the use of different tools or the differences in participants’ characteristics. In the study by Bartnik et al. (2017), the evaluation of SD was limited to patients with Relapsing-Remitting MS (RRMS), and in the study by Lew-Starowics (2013), the mean disability score was 5.2, which differs from the score obtained in the present study [1, 32].

In the present study, the highest prevalence of SD pertained to sexual desire, arousal and not reaching orgasm, in respective order, which agrees with the results obtained by Lew-Starowics (2013) [32]. Also, In a meta-analysis study by Ranjbaran (2016), the overall prevalence of SD in Iranian women without MS was 43.9% and prevalence of desire, arousal, lubricating, orgasmic, satisfaction and pain disorders were 42.7% (32.0–53.4), 38.5% (27.6–49.5), 30.6% (22.0–39.2), 29.2% (24.1–34.3), 21.6% (11.5–31.8) and 40.1% (31.8–48.3), respectively [36]. The most common sexual problems reported in most studies were dysfunctional sexual desire and arousal, which are mainly due to the psychological state in women, while lubrication and intercourse problems are mainly caused by physiological disorders. The psychological complications of MS appear to have greater effects than physiological complications on women’s sexual function [37].

In the present study, an inverse and significant correlation was observed between age and the total score of FSFI and its subscale scores, which agrees with the results obtained by Merghati-Khoei’s study (2014). Gumus(2014), Gava(2019) also found a negative correlation between total FSFI score and age in MS women. Nonetheless, the results obtained by Ghajarzadeh (2014) and Mohammadi (2013) showed no relationships between age and SD. Also, No significant relationships were found in Bartnik (2017) study between the total FSFI score and age, but a significant negative correlation was observed only between age and the desire score (*P* = 0.02, *r* = − 0.26), and a significant positive correlation between age and the pain score(*P* = 0.02, *r* = 0.26), which agrees with the results obtained in the present study. Moreover, an inverse and significant correlation was found between the total scores of FSFI and its subscale scores and the duration of marriage, which could be due to the changes in the perception of problems and their abating as a result of less frequent sexual activity over time. Merghati-Khoei (2014) also found an inverse correlation between SD and duration of marriage, but Alehashemi (2019) observed no such relationships. This disparity in findings may be due to the small sample size in the aforementioned study (*n* = 64) as well as the differences in study type (case-control). A negative and significant correlation was found in the present study between fatigue and the total FSFI score and its subscale scores. In Bartnik’s study (2017), SD was mildly and negatively correlated with fatigue in women with RRMS, and the severity of fatigue had a negative effect on sexual function (*P* <  0.05). Severe fatigue is a typical sign of MS that is associated with secondary SD [32]. Women’s sexual disorders are more prevalent in patients with multiple sclerosis (MS), even in early disease stages when severe disability is absent [38] which is significantly more than in general population samples [39] or healthy controls [40]. In the present study, a negative and significant correlation was found between the EDSS score and the total scores of FSFI and its subscales, except for desire and pain, and studies by Alehashemi (2019) and Ghajarzadeh (2014) showed a significant negative relationship between the EDSS scores and the total FSFI scores (*P* = 0.032, *r* = 0.35 and *P* = 0.001, *r* = 0.44). Koranian (2017) also found an inverse and significant relationship between the EDSS level and the total FSFI score and its subscale scores, except for pain (*P* = 0.001, *r* = 0.61), and Konstantinidis (2019) also found an inverse and significant relationship between the EDSS level and the FSFI subscale scores, except for arousal and satisfaction [41]. Celik (2013) showed a direct and significant relationship between the EDSS score and secondary (*P* = 0.001, *r* = 0.34) and tertiary (*P* = 0.032, *r* = 0.35) SD. Additionally, Gumus (2014), Gava (2019) and Zivadinov (2003) also found a negative correlation between total FSFI score and EDSS in MS women [38, 39, 42].. In Bartnik’s study (2017), however, the self-reported EDSS scores had no relationship with the FSFI subscale scores. In the present study, age at diagnosis had inverse correlations with the arousal and pain scores. Bartnik (2017) showed a negative and significant relationship between age at diagnosis and the total score of FSFI (*P* <  0.02, *r* = 0.27), but is not consistent with the results of Merghati-Khoei et al. (2013) study. This could be attributed to the different questionnaire (MSISQ-19).

A study focusing on sexual dysfunctions in chronic diseases found that comorbid depressionwas frequent and independently determined the presence of sexual dysfunction [43]. In the present study, a negative and significant correlation was found between the depression score and the total scores of FSFI and its subscales, except for desire, lubrication, and pain, and studies by Gumus et al. (2014) and Ghajarzadeh (2014) showed a significant negative relationship between the depression scores and the total FSFI scores (*P* = 0.032, *r* = 0.35 and *P* = 0.001, *r* = 0.44).Young et al.(2016) investigated SD in relation to depression, fatigue and physical function in 538 people with MS and found no direct association between depression and sexual functioning, but depression appeared as a consequence of the psychological issues associated with SD [44]. In the Ashtari et al. (2014) study, high frequency of women with tertiary SD and strong relationship between primary and tertiary SD emphasized the effective role of psychosocial factors on sexual function of MS women in different levels.Depression in MS is a multidimensional problem that varies by disease related impairments, activity restrictions and unpredictable prognosis. Also, in patients with MS it is significantly higher than healthy individuals and is considered as a common co-morbidity among patients with multiple sclerosis [8]. No significant relationships were found in the present study between the total FSFI score and disease duration, and a significant negative correlation was observed only between disease duration and the lubrication scores, which agrees with the results obtained by Bartnik et al. (2017) and Alehashemi (2019), although Celik (2013) found a direct significant relationship between secondary SD and disease duration (*P* = 0.042, *r* = 0.21). In the present study, no significant differences were observed between the three groups in the mean scores of FSFI and its subscale scores (except for lubrication) in terms of the clinical course of MS. Similarly, no significant differences were found between SD and the clinical course of the disease in Ashtari’s (2014), Zivadinov (1999) studies. In Demirkiran’s study (2006), however, a correlation was observed between SD and the progressive form of the disease [45] The results of one study showed that the likelihood of SD is three times higher in progressive MS compared to RRMS [8] In the present study, no significant differences were observed between the three groups in the mean scores of FSFI and its subscale scores (except for Arousle) in terms of the education. This is in line with some studies [12, 46], but is not consistent with the results of study [1, 8]. Moreover, logistic regression analysis revealed that there was a higher probability of a sexual dysfunction among patients with MS and a high fatigue score [1.228(1.003 to 1.504); *P* = 0.047]. In the present study, the mean scores of all the MSQOL subscales were higher in the married women with MS and SD compared to those without SD, and the differences between the two groups were significant in all the dimensions, except for role limitation due to mental problems and change in health. In studies conducted by Qaderi (2014) and Tepavccvic (2008), a negative and significant relationship was observed between most dimensions of MSQOL (such as physical health, role limitation due to physical problems, social function and cognitive function) and SD [13, 47]. In the present study, a significant difference was found between the women with and without SD in terms of the physical and mental health composites, which agrees with the results obtained by Schairer (2014) and Vitkova (2014), which suggested a negative and significant relationship between SD and the physical and mental dimensions of MSQOL [48, 49]. The limitations of this study included the subjects’ memory capacity in recalling past information as well as the questioner’s presence during questionnaire completion, which could have affected the subjects’ responding to the questionnaire items. Another limitation of the present study have not been asked information about issues such as menopause; contraceptive use. In addition, hormonal evaluation could not be applied to patients.

## Conclusion

Sexual dysfunction is highly prevalent among women with MS and affects different aspects of quality of life. Fatigue is important predictor of SD in these patients. Improving quality of life in this group seems to require greater attention to their sexual problems. Sexuality is a two-sided issue and MS also impacts upon other members of patient’s family. Therefore, investigation of sexuality in patients’ partners was suggested for future research.

## Data Availability

Not applicable.

## Abbreviations

EDSS: Expanded disability status scale
IPSS: International prostate symptom score
LUTS: Lower urinary tract symptoms
MRI: Magnetic resonance imaging
MS: Multiple sclerosis
MSAA: Multiple Sclerosis Association of America
QOL: Quality of life
QOL: Quality of life
RRMS: Relapsing-Remitting MS
SPMS: Secondary progressive MS
SUI: Stress urinary incontinence
